# Supplementary material for: FedLGAN: a method for anomaly detection and repair of hydrological telemetry data based on federated learning
Source: PeerJ Comput Sci. 2023 Nov 7;9:e1664. doi: 10.7717/peerj-cs.1664 (PMC10703019; doi:10.7717/peerj-cs.1664)
Supplement: Supplemental Information 1 [file peerj-cs-09-1664-s001.docx]

The code is available at GitHub: <https://github.com/2450848351/FedLGAN> or at Zenodo: [DOI: 10.5281/zenodo.8286185](https://doi.org/10.5281/zenodo.8286185)

The partially processed hydrological telemetry data is available at GitHub: <https://github.com/2450848351/FedLGAN/tree/master/data> or at Zenodo: [DOI: 10.5281/zenodo.8286185](https://doi.org/10.5281/zenodo.8286185)
